# Parametric Sensitivity of Shear Correction Factors for Multiwall Corrugated Structures

**DOI:** 10.3390/ma19050863

**Published:** 2026-02-26

**Authors:** Julia Graczyk, Jędrzej Tworzydło, Tomasz Garbowski

**Affiliations:** 1 Institute of Structural Analysis, Poznan University of Technology, 60-965 Poznan, Poland; julia.graczyk@student.put.poznan.pl; 2 Werner Kenkel Bochnia Spółka z o. o., 32-700 Bochnia, Poland; jedrzej.tworzydlo@wernerkenkel.com.pl; 3 University Center for Eco-materials, Poznan University of Life Sciences, 60-637 Poznan, Poland

**Keywords:** corrugated board, shear correction factor, transverse shear deformation, pixel-based modeling, global sensitivity analysis, Latin hypercube sampling

## Abstract

Transverse shear deformation plays a non-negligible role in lightweight periodic-core structures and motivates the use of shear-corrected reduced-order plate and beam models. However, the shear correction factor $k_{s}$ is often treated as a constant despite its strong dependence on cross-sectional heterogeneity and geometry. This work quantifies the global sensitivity of $k_{s}$ in corrugated paperboard by combining an energy-consistent pixel-based identification of the effective shear stiffness $\left(GA{)}_{eff}\right.$ with a space-filling exploration of the parameter domain. Representative three-ply (single-wall) and five-ply (double-wall) configurations are generated directly in the pixel domain using sinusoidal fluting descriptions and non-overlapping liner bands. The effective shear stiffness is obtained from a heterogeneous shear-energy equivalence, where a normalized two-dimensional shear-stress shape function is computed from pixel-based sectional descriptors and integrated with spatially varying shear moduli. Latin Hypercube Sampling is employed to explore wide ranges of flute period, height, and thickness, liner thicknesses, and liner–flute shear-modulus contrasts. Global sensitivity is reported using unit-free normalized indices, including log-elasticities (based on the slope of $\ln k_{s}$ versus lnx) and partial rank correlation coefficients. The results demonstrate that flute geometry is the primary driver of $k_{s}$ variability, while material contrast significantly modulates shear-energy localization, particularly in double-wall boards with two distinct flutings. The proposed framework enables high-throughput shear correction assessment and supports robust parameterized reduced-order models for corrugated structures.

## 1. Introduction

Lightweight layered and cellular structures are widely used in engineering whenever high stiffness-to-weight and controllable mechanical response are required. Typical examples include sandwich panels, rib-stiffened shells, corrugated cores, honeycomb configurations, and architected metamaterials. In many applications, the global structural response is governed not only by material properties but also by geometry-driven mechanisms such as local bending of webs, shear deformation within compliant cores, and stress redistribution around voids. As a result, accurate analysis and design require modeling frameworks that can connect microstructural geometry to macroscopic stiffness and strength while remaining computationally efficient for parametric studies and optimization tasks [1,2,3,4,5].

A common strategy in this context is to replace a detailed heterogeneous configuration with a homogenized or equivalent continuum model. For layered and periodic structures this often takes the form of an equivalent orthotropic plate or beam, typically derived using energy-based homogenization or computational representative volume elements (RVEs) [6,7,8,9,10,11,12,13]. While such reduced-order models offer major computational advantages, their accuracy depends critically on how they represent the internal deformation mechanisms of the original structure. Among these mechanisms, transverse shear is particularly challenging: it is negligible for thin homogeneous plates but becomes decisive for thick laminates, soft-core sandwich structures, and periodic cores with voids or inclined webs [14,15,16,17,18,19,20].

In classical plate theory, shear deformation is neglected (Kirchhoff–Love model), leading to good predictions for slender plates dominated by bending. In contrast, in the Timoshenko–Mindlin family of models, transverse shear is included and a key parameter appears: the shear correction factor $k_{s}$. This factor compensates for the fact that the assumed through-thickness shear strain (often constant) does not match the true shear stress distribution, which depends on cross-sectional geometry and material heterogeneity. In homogeneous rectangular beams and plates, reference values of $k_{s}$ are available (e.g., the well-known factors for rectangular cross-sections). However, for strongly heterogeneous, perforated, or architected cross-sections, $k_{s}$ cannot be assumed universal and must be identified consistently [21,22,23,24,25,26].

The need for reliable shear correction is especially evident in periodic cores and multi-layered configurations. For corrugated or honeycomb-like microstructures, shear stresses may localize in thin load paths, bypass void regions, and be redistributed by stiff faces. In such systems, the effective shear response is governed by an interplay of geometry (period, height, thickness, topology) and phase stiffness contrast. Consequently, inaccurate shear correction can lead to systematic errors in predicted deflections, stress resultants, and even in derived design conclusions when simplified plate models are used for parametric design [27,28,29].

A second challenge is that shear correction factors are frequently introduced in reduced-order models in a way that is not energy-consistent, especially when one attempts to reuse simple textbook factors for cross-sections with complex heterogeneity. Energy-consistent identification is crucial because the effective shear stiffness must reproduce not only the global deflection but also the correct shear energy stored in the structure under transverse loading. For heterogeneous layered systems, energy-based equivalence provides a robust path to define the effective shear stiffness $\left(GA{)}_{eff}\right.$ and to derive $k_{s}$ relative to an explicit reference model [10,26].

A practical difficulty remains: even if an energy-consistent definition is adopted, computing $\left(GA{)}_{eff}\right.$ repeatedly for many design variants can be expensive, particularly if full-scale finite element simulations are required for each geometric configuration. This becomes a bottleneck when the goal is not a single calibration but a systematic assessment of how sensitive $k_{s}$ is to changes in geometry and material properties across a realistic design space. Such sensitivity knowledge is important in at least three contexts: (i) robust design under manufacturing tolerances and paper variability, (ii) the development of fast surrogate models for design optimization, and (iii) deciding which parameters must be measured and controlled to ensure predictive reliability of reduced-order models [30,31,32].

Corrugated board is a representative and industrially relevant example of such a geometry-dominated, heterogeneous structure. It consists of flat paper liners bonded to one or more sinusoidally shaped fluting layers, forming 3-ply (single-wall) and 5-ply (double-wall) assemblies commonly used in packaging. Corrugated board is simultaneously lightweight, recyclable, and mechanically efficient, yet its response is strongly influenced by flute geometry, paper anisotropy, and moisture-dependent stiffness. Because its internal architecture contains voids and inclined webs, transverse shear deformation may contribute significantly to certain test configurations and structural applications, especially at short spans and under out-of-plane loading [33,34,35,36,37].

A substantial body of research addresses bending stiffness, edge crush strength, and constitutive modeling of corrugated board under various loading conditions. Reduced-order modelling approaches, including homogenized orthotropic plates, are widely used to predict global stiffness and to support package design [38,39]. Nevertheless, compared to bending-dominated metrics, the identification and systematic characterization of shear correction remains less developed, particularly for multiwall boards and for broad variations in geometry and material stiffness that arise across flute families and paper grades.

In addition, sensitivity studies reported in the literature often focus on local effects (small perturbations around a baseline configuration) or on a limited set of parameters. While such analyses can be informative, they do not necessarily capture the full range of coupled effects present in corrugated structures. When a response is governed by nonlinear geometric interactions, void topology, and stiffness contrast, the ranking of influential parameters may change across the domain. In this case, global sensitivity analysis, based on space-filling sampling strategies such as Latin Hypercube Sampling (LHS), provides a more appropriate framework to evaluate parameter influence, detect interactions, and identify robust trends [31,40].

Motivated by these gaps, the present work investigates the sensitivity of the shear correction factor $k_{s}$ for corrugated boards across a broad multi-parameter space, focusing on both three-ply and five-ply configurations. The study adopts a pixel-based, energy-consistent procedure for computing $\left(GA{)}_{eff}\right.$ directly from the cross-sectional geometry and spatially varying shear modulus fields, avoiding ad hoc assumptions and enabling high-throughput evaluation [41,42,43,44,45,46,47]. The parameter space is explored using LHS, and sensitivity is quantified in a normalized, unit-free manner using measures that directly express percentage-to-percentage influence, enabling an interpretable ranking of drivers across variables with different physical dimensions [10,35].

The main contributions of this paper are: (a) a global (nonlocal) sensitivity assessment of $k_{s}$ for corrugated paperboard performed over a wide admissible parameter space, rather than relying on local perturbations around a single reference design; this reveals dominant drivers and interactions relevant for real product families and manufacturing variability; (b) a unified framework for three-ply and five-ply boards, explicitly allowing distinct geometries and stiffnesses of two flutings in the double-wall case, which is essential for capturing multi-layer coupling effects that cannot be inferred from single-wall trends; (c) an energy-consistent, pixel-based evaluation of $\left(GA{)}_{eff}\right.$ enabling high-throughput computation of $k_{s}$ across thousands of sampled designs, offering a practical route toward surrogate modeling, robust design, and reliable shear-corrected reduced-order models; (d) normalized, unit-free sensitivity indices (%/%), based on classical log-elasticities and complementary Partial Rank Correlation Coefficients (PRCC), employed to provide directly interpretable percentage-to-percentage influence measures.

These outcomes are important because they help transform $k_{s}$ from a loosely chosen tuning factor into a geometry- and material-dependent quantity with quantified uncertainty and sensitivity, improving the reliability of reduced-order models used in packaging mechanics and other periodic-core structural applications. The proposed framework enables a global sensitivity assessment across a broad admissible parameter space; it does not constitute a formal probabilistic uncertainty quantification framework but rather evaluates parameter influence and variability trends.

## 2. Materials and Methods

### 2.1. The Scope of the Study and Investigated Configurations

This study quantifies the sensitivity of the transverse shear correction factor $k_{s}$ to the key geometric and material parameters of corrugated paperboard. Two structural families are considered: (i) 3-ply (single-wall): top liner + sinusoidal fluting + bottom liner; (ii) 5-ply (double-wall): top liner + fluting 1 + middle liner + fluting 2 + bottom liner.

For the double-wall configuration, the two flutings may exhibit different periods, heights and thicknesses, enabling a systematic evaluation of geometric interactions. The analysis is conducted over a wide admissible parameter space using global sampling (Latin Hypercube), which allows for the identification of dominant parameters and parameter couplings beyond local perturbations around a single reference design. All layers are assumed to be perfectly bonded, and interfacial contact or slip effects are not considered in the present linear-elastic framework.

### 2.2. Definition of the Shear Correction Factor

The shear correction factor is defined by comparing the effective transverse shear stiffness of the heterogeneous cross-section with the shear stiffness of a flat reference model:

(1) $$k_{s}=\frac{\left(GA{)}_{eff}\right.}{G^{*}A^{*}}.$$

The reference area is taken as

(2) $$A^{*}=b\;H,$$

where b denotes the out-of-plane width (unit width is adopted in computations) and H is the adopted reference height corresponding to the total thickness of the considered corrugated board configuration.

To ensure a stable normalization across broad geometric and material variations, a thickness-weighted reference shear modulus is used:

(3) $$G^{*}=\sum_{k=1}^{n_{l}}w_{k}\;G_{k},w_{k}=\frac{t_{k}}{\sum_{j=1}^{n_{l}}t_{j}},$$

where $t_{k}$ and $G_{k}$ are the thickness and shear modulus of the k-th layer (liners and flutings). The thickness-weighted reference modulus provides a neutral normalization across heterogeneous configurations. Alternative reference definitions would scale the numerical value of $k_{s}$ but would not alter relative sensitivity rankings. This definition avoids imposing a priori that only one phase carries shear and ensures consistent comparison between 3-ply and 5-ply configurations.

### 2.3. Parametric Geometry and Pixel-Based Representation

#### 2.3.1. Sinusoidal Fluting Geometry

The fluting centerline is defined by:

(4) $$z(x)=z_{0}+\frac{h}{2}cos\left(\frac{2\pi }{p}x+\varphi \right),$$

with period p, height h (peak-to-peak), vertical offset $z_{0}$, and phase shift φ. Finite thickness $t_{f}$ is introduced by constructing a constant-thickness band around the centerline. Although real corrugated boards may exhibit minor deviations from ideal sinusoidal profiles, the adopted representation captures the dominant geometric features and can be readily extended to arbitrary centerline shapes within the pixel-generation framework.

For 5-ply boards, two flutings are described independently by $\left(p_{1},h_{1},t_{f1}\right)$ and $\left(p_{2},h_{2},t_{f2}\right)$ and are placed into two non-overlapping core bands between (top, middle) and (middle, bottom) liners, respectively.

#### 2.3.2. Pixel Image Generation and Phase Encoding

The cross-section is represented by a grayscale pixel image generated in MATLAB. (R2024a) The following encoding is used:

- Background (void): intensity 1.0,
- Liners: intensity 0.5,
- Fluting(s): intensity 0.0.

The physical pixel size pix (mm/pixel) controls the geometric resolution. Within the investigated industrial parameter ranges, this constraint does not exclude realistic configurations but ensures numerical stability of thin-layer representation. To prevent the loss of thin layers and boundary artifacts, the admissible domain is constrained, such that:

(5) $$t_{min}\geq 2\;pix,$$

where $t_{min}$ is the minimum thickness among all considered liners and flutings. The same generator is employed both for methodological figures and for the numerical evaluation of $k_{s}$, ensuring full consistency between geometry visualization and computation.

### 2.4. Pixel-Based Identification of $\left(GA{)}_{eff}\right.$

#### 2.4.1. Energy-Consistent Formulation

The effective transverse shear stiffness is identified by enforcing equivalence between the shear strain energy of a flat Timoshenko model and the heterogeneous cross-section under the same shear resultant V. For a representative segment of length L, the flat-model energy reads:

(6) $$U_{flat}=\frac{1}{2}\;\frac{V^{2}}{\left(GA{)}_{eff}\right.}\;L.$$

For a heterogeneous cross-section, the shear energy may be expressed using a normalized 2D shear-stress shape function ϕ(x,z) defined such that:

(7) $$\tau_{xz}(x,z)=V\;\phi (x,z),\int_{A}\phi (x,z)\;dA=1,$$

which guarantees that integration of $\tau_{xz}$ over the cross-section and yields the shear resultant V. Physically, ϕ(x,z) represents the normalized shear stress distribution associated with a unit shear resultant, ensuring equilibrium and energy equivalence between the heterogeneous cross-section and its reduced-order representation. Under linear elasticity, the heterogeneous shear energy becomes:

(8) $$U_{pix}=\frac{1}{2}\int_{A}\frac{\tau_{xz}^{2}(x,z)}{G(x,z)}\;dA=\frac{1}{2}V^{2}\int_{A}\frac{\phi^{2}(x,z)}{G(x,z)}\;dA.$$

By imposing $U_{flat}=U_{pix}$, the effective shear stiffness is obtained as:

(9) $$(GA{)}_{eff}={\left(\int_{A}\frac{\phi^{2}(x,z)}{G(x,z)}\;dA\right)}^{-1}.$$

Finally, $k_{s}$ follows from Equation (1).

#### 2.4.2. Pixel Discretization and Material Mapping

The cross-section is discretized into pixels (or subpixels) of area ΔA. Void pixels are excluded from the mechanical domain. Each material pixel i is assigned a shear modulus $G_{i}$ based on its phase label (liner vs. fluting; in 5-ply cases, fluting 1 and fluting 2 may have different G). This yields a discrete material field $G(x,z)\to G_{i}$ and a corresponding set of integration weights $\Delta A_{i}$. The influence of pixel resolution on effective shear stiffness is assessed in Section 3.1, confirming numerical stability under refinement.

#### 2.4.3. Construction of the 2D Shear-Stress Shape Function ϕ(x,z)

To avoid introducing additional modeling assumptions beyond geometry and material phase distribution, the shear-stress shape function ϕ(x,z) is constructed directly from the pixelized cross-section using a geometry-consistent 2D formulation. The approach relies on the classical relationship between shear stresses and the first moment of area (static moment) while retaining full 2D resolution required by corrugated geometries.

First, the area and centroid of the material domain are computed from pixel quadrature:

(10) $$A=\int_{A}1\;dA\approx \sum_{i=1}^{N_{p}}\Delta A_{i},\;\;\bar{z}=\frac{1}{A}\int_{A}z\;dA\approx \frac{1}{A}\sum_{i=1}^{N_{p}}z_{i}\;\Delta A_{i}.$$

Next, the second moment of area about the centroidal axis is evaluated:

(11) $$I=\int_{A}(z-\bar{z}{)}^{2}\;dA\approx \sum_{i=1}^{N_{p}}(z_{i}-\bar{z}{)}^{2}\;\Delta A_{i}.$$

To define the shear-stress distribution across the cross-section, a 2D static-moment field Q(x,z) is introduced. For a point $\left(x,z\right)$ in the material domain, Q(x,z) represents the first moment of the area of the portion of the cross-section located above that point (with respect to the centroidal axis). In discrete form, for every material pixel i at coordinates $\left(x_{i},z_{i}\right)$, we define:

(12) $$Q_{i}\equiv Q(x_{i},z_{i})=\int_{A(z\geq z_{i})}(z-\bar{z})\;dA\approx \sum_{\begin{array}{c} j=1\\ z_{j}\geq z_{i} \end{array}}^{N_{p}}(z_{j}-\bar{z})\;\Delta A_{j}.$$

The local thickness measure entering the shear-stress relation is defined from the pixel domain as an effective local width b(x,z), computed as the material measure in the horizontal direction associated with the given pixel row. In the discretized setting, this can be expressed as:

(13) $$b(z_{i})\approx \frac{1}{\Delta z}\sum_{\begin{array}{c} j=1\\ z_{j}\in [z_{i}-\Delta z/2,z_{i}+\Delta z/2] \end{array}}^{N_{p}}\Delta A_{j},$$

which corresponds to the length of material present at the level $z=z_{i}$ (for unit out-of-plane width). Since Δz corresponds to the pixel height, its influence is governed by the same resolution considerations discussed in Section 3.1; within the investigated range, no significant bias in the resulting shape function was observed. This definition naturally captures the fact that corrugated sections may be discontinuous along x at a given height due to void regions.

With these ingredients, the unnormalized shear-stress shape field is computed as:

(14) $${\tilde{\phi }}_{i}=\frac{Q_{i}}{I\;b(z_{i})}.$$

Finally, the field is normalized to satisfy the resultant condition in Equation (7):

(15) $$\phi_{i}=\frac{{\tilde{\phi }}_{i}}{\sum_{k=1}^{N_{p}}{\tilde{\phi }}_{k}\;\Delta A_{k}}.$$

Equations (12)–(15) define a fully 2D, geometry-consistent shape function computed directly from the pixel representation. This construction avoids prescribing shear localization a priori and allows the subsequent energy evaluation to reflect both geometric complexity (corrugation) and spatially varying shear modulus G(x,z).

#### 2.4.4. Discrete Evaluation of $\left(GA{)}_{eff}\right.$ and $k_{s}$

With $\phi_{i}$ and $G_{i}$ defined on material pixels, Equation (9) becomes:

(16) $${\left(GA\right)}_{eff}={\left(\sum_{i=1}^{N_{p}}\frac{\phi_{i}^{2}}{G_{i}}\;\Delta A_{i}\right)}^{-1}.$$

The shear correction factor is then computed from Equation (1):

All computations are performed for a unit width b=1, and the reported values of $k_{s}$ are therefore independent of scaling in the out-of-plane direction. The consistency of the present energy-based identification with classical analytical shear correction factors for homogeneous rectangular sections has been verified in our previous works [41,42,43,44], where convergence toward the reference value $k_{s}=5/6$ was demonstrated.

### 2.5. Global Sensitivity Analysis Using Latin Hypercube Sampling

#### 2.5.1. Parameter Vectors

For the 3-ply configuration, the parameter vector is:

(17) $$x_{3}=\left[p,\;h,\;t_{f},\;t_{L,t},\;t_{L,b},\;G_{f},\;G_{L}\right].$$

For the 5-ply configuration:

(18) $$x_{5}=\left[p_{1},h_{1},t_{f1},\;p_{2},h_{2},t_{f2},\;t_{L,t},t_{L,m},t_{L,b},\;G_{f1},G_{f2},G_{L}\right].$$

This parametrization enables the separation of geometric and material influences and explicitly allows different flute families in the double-wall board.

#### 2.5.2. Parameter Ranges and Feasibility Constraints

The selected geometric ranges correspond to representative industrial flute families (e.g., E-, B-, and C-type boards), while the shear modulus intervals reflect commonly reported variability of paper grades and moisture-dependent stiffness values. The admissible ranges are selected to cover representative corrugated geometries and material variability while ensuring stable discretization:

Three-ply:

- $p\in \left[4.0,\;8.5\right]\;$ mm,
- $h\in \left[1.0,\;4.5\right]\;$ mm,
- $t_{f}\in [0.10,\;0.35]$ mm,
- $t_{L,t},t_{L,b}\in \left[0.10,\;0.40\right]\;$ mm,
- $G_{f}\in \left[0.15,\;1.50\right]\;$ GPa,
- $G_{L}\in \left[0.25,\;2.50\right]\;$ GPa.

Five-ply:

- $p_{1},p_{2}\in [4.0,\;9.5]$ mm,
- $h_{1},h_{2}\in \left[1.0,\;5.0\right]\;$ mm,
- $t_{f1},t_{f2}\in \left[0.10,\;0.35\right]\;$ mm,
- $t_{L,t},t_{L,m},t_{L,b}\in \left[0.10,\;0.40\right]\;$ mm,
- $G_{f1},G_{f2}\in \left[0.15,\;1.50\right]\;$ GPa,
- $G_{L}\in \left[0.25,\;2.50\right]\;$ GPa.

All samples must satisfy: (i) no overlap of phases (enforced by construction in the image generator), and (ii) the resolution condition in Equation (5).

#### 2.5.3. LHS Strategy

A Latin Hypercube Sampling (LHS) design of size N is generated for each configuration family. Geometric variables are sampled from uniform distributions within their bounds. All input variables are sampled independently within the admissible domain; no statistical correlations are imposed a priori. Observed coupling effects therefore arise from the mechanical response of the structure rather than from predefined input dependencies. To represent multiplicative uncertainty in stiffness, shear moduli are sampled from a log-uniform distribution:

(19) $$\log G∼U\left(\log G_{min},\log G_{max}\right).$$

For each sampled vector $x^{\left(n\right)}$, the computational pipeline is:

1. generate pixel geometry,
2. assign shear modulus field $G_{i}$,
3. compute the 2D shape function $\phi_{i}$ using Equations (12)–(15),
4. evaluate $\left(GA{)}_{eff}\right.$ by Equation (16),
5. compute $k_{s}$ by Equation (1).

This produces the dataset $\left\{(x^{\left(n\right)},k_{s}^{\left(n\right)}){\}}_{n=1}^{N}\right\}$ used in subsequent analysis.

#### 2.5.4. Normalized Global Sensitivity Metrics

To quantify sensitivity in a unit-free and comparable manner across variables, two complementary normalized indices are adopted. First, we use log-elasticities, defined as

(20) $$S_{j}=\frac{\partial \ln k_{s}}{\partial \ln x_{j}},$$

which directly represent the percentage change of $k_{s}$ per percentage change in the input variable $x_{j}$. In practice, $S_{j}$ is estimated by fitting a linear model in logarithmic space,

(21) $$\ln k_{s}=\beta_{0}+\sum_{j=1}^{d}\beta_{j}\ln x_{j}+\varepsilon ,$$

so that $S_{j}\approx \beta_{j}$. Second, we compute partial rank correlation coefficients (PRCC), which quantify the influence of each variable while controlling for the remaining inputs and are robust to monotonic nonlinearities. The log-elasticity measure corresponds to the classical definition of elasticity in logarithmic space and is widely used in global sensitivity analysis; it is not a newly introduced metric but an established interpretation of multiplicative sensitivity. Both indices are evaluated on the dataset generated by Latin Hypercube Sampling and are reported as normalized, dimensionless sensitivity measures.

### 2.6. Computational Implementation and Reproducibility

All calculations are performed in MATLAB. Geometry generation and pixel-based integration are fully automated to enable high-throughput evaluation of $k_{s}$ across LHS samples. For stochastic designs, a fixed pseudo-random seed is used to ensure reproducibility. A mesh-resolution robustness check is conducted on a representative subset of samples to confirm that the computed $k_{s}$ values and sensitivity trends are not affected by pixel discretization artifacts. The underlying energy-consistent identification procedure has previously been validated against shell- and solid-based finite element simulations for heterogeneous cross-sections, including corrugated configurations [10,41,43,47]. The present study therefore focuses on large-scale parametric sensitivity rather than repeated FE validation.

## 3. Results

### 3.1. Dataset Overview and Numerical Stability

Global datasets for three-ply and five-ply corrugated boards were generated using Latin Hypercube Sampling (LHS) within the prescribed geometric and material parameter ranges summarized in Table 1. For each LHS realization, the corresponding geometry was constructed directly in the pixel domain, and the shear correction factor $k_{s}$ was computed using the pixel-based energy-consistent method described in Section 2. After enforcing feasibility and minimum-resolution constraints, the final datasets consisted of $N_{3}$ valid samples for the three-ply configuration and $N_{5}$ samples for the five-ply configuration.

**Figure 1:**
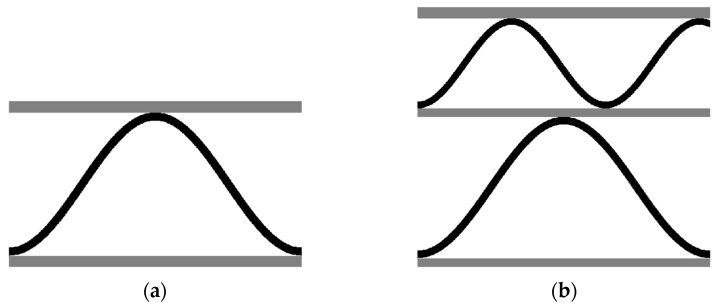
Pixel-based geometry examples generated for the sensitivity study: (**a**) 3-ply reference geometry and (**b**) 5-ply example with two distinct flutings. Liners are shown in gray, fluting layers in black, and void regions in white. The vertical axis corresponds to board thickness, and pixel intensities represent the material phase assignment within the cross-section.

Representative examples of the pixel-based geometries generated within these ranges are shown in Figure 1, illustrating the fundamental structural differences between the single-flute three-ply board and the double-flute five-ply configuration.

To verify the numerical robustness of the pixel discretization, a resolution sensitivity study was performed on a representative subset of samples by repeating the computations using a refined pixel size. The relative changes in the shear correction factor, $\Delta k_{s}/k_{s}$, are summarized in Table 2.

The limited magnitude of these variations confirms that the global trends discussed in the following sections are not governed by discretization artifacts but reflect genuine geometric and material effects.

### 3.2. Distribution of the Shear Correction Factor $k_{s}$

**Figure 2:**
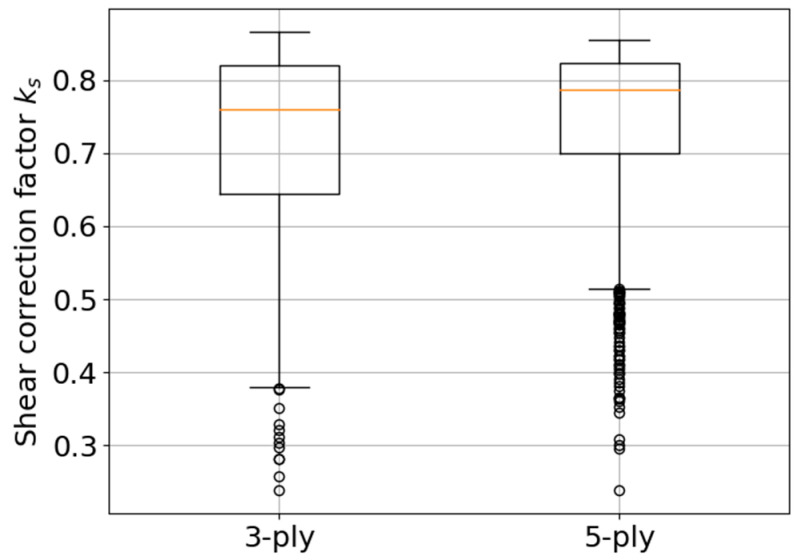
Distribution of $k_{s}$ obtained from LHS: 3-ply and 5-ply. The histogram/violin representation highlights the spread, skewness, and presence of tails corresponding to extreme geometries or stiffness contrasts.

Across the investigated parameter space, the computed shear correction factors exhibit a pronounced spread, reflecting the strong dependence of transverse shear behavior on both geometry and material contrast. The empirical distributions obtained from the LHS datasets for the three-ply and five-ply configurations are shown in Figure 2.

While the three-ply boards exhibit a relatively compact distribution, the five-ply boards display a markedly wider dispersion and heavier tails, as evident in Figure 2. The broader distribution observed for five-ply configurations indicates that additional structural degrees of freedom amplify shear response variability. A quantitative summary of these distributions is provided in Table 3.

### 3.3. Global Sensitivity: Normalized Effects (%/%)

To quantify parameter influence in a unit-free and directly interpretable manner, two complementary global sensitivity measures were employed: (i) log-elasticities (Equation (20)) and (ii) Partial Rank Correlation Coefficients (PRCC).

The resulting normalized sensitivity indices for the three-ply and five-ply configurations are reported in Table 4 and Table 5, respectively.

The magnitude of the log-elasticity associated with flute height confirms its dominant influence, exceeding that of thickness and material parameters by a clear margin.

**Figure 3:**
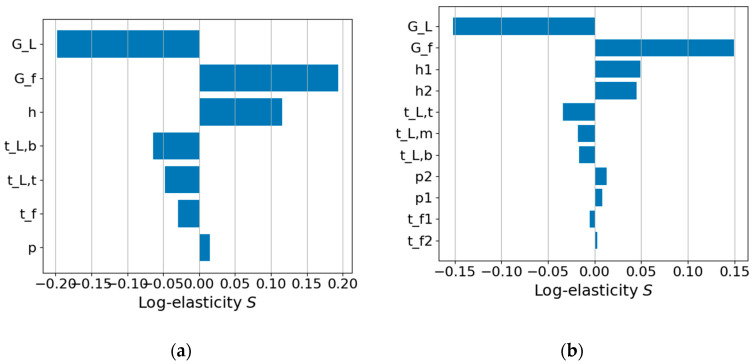
Tornado plots of normalized sensitivities (log-elasticities) ranked by absolute magnitude: (**a**) 3-ply and (**b**) 5-ply. This figure provides an immediate ranking of the dominant drivers of $k_{s}$ across the global parameter space.

In the five-ply case, both fluting layers exhibit comparable influence, confirming that shear behavior is governed by distributed geometric mechanisms rather than a single dominant layer. For a concise visual ranking of the dominant drivers, the log-elasticities are displayed in the tornado plots shown in Figure 3.

### 3.4. Response Surfaces for the Dominant Geometric Drivers

**Figure 4:**
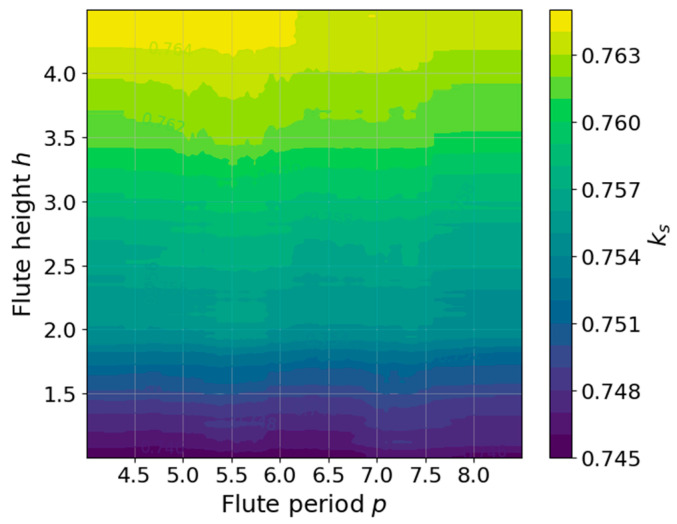
2D response surface for 3-ply: $k_{s}(p,h)$ at median values of $\left(t_{f},t_{L,t},t_{L,b},G_{f},\;G_{L}\right)$. Contours indicate regions of high and low $k_{s}$.

For the three-ply configuration, the response surface $k_{s}(p,h)$ evaluated at median values of the remaining parameters is shown in Figure 4.

**Figure 5:**
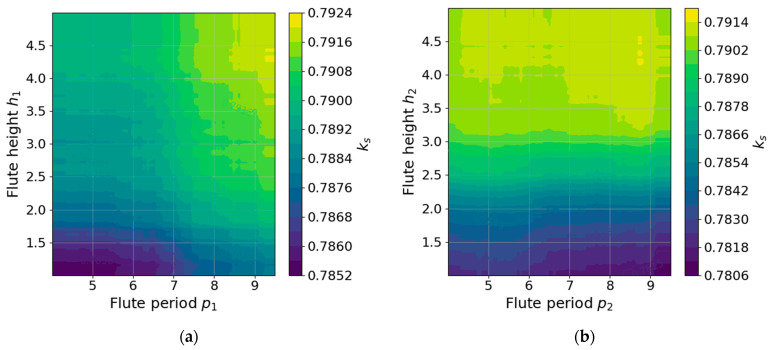
2D response surfaces for 5-ply: (**a**) $k_{s}(p_{1},h_{1})$ with the second flute fixed at median parameters; (**b**) $k_{s}(p_{2},h_{2})$ with the first flute fixed. These maps highlight how each flute family contributes to the global shear correction behavior.

Analogous response surfaces for the five-ply configuration are presented in Figure 5, where each fluting family is examined separately.

### 3.5. Material Contrast Effects and Key Interactions

**Figure 6:**
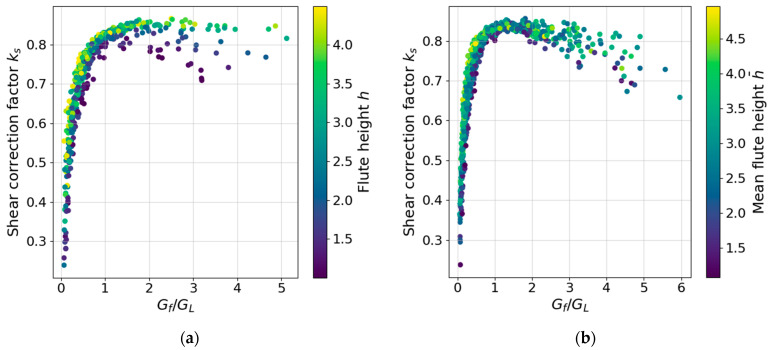
Effect of shear-modulus contrast: (**a**) scatter of $k_{s}$ versus $G_{f}/G_{L}$(3-ply) and (**b**) versus $\left(G_{f1}/G_{L},\;G_{f2}/G_{L}\right)$ (5-ply), with points colored by a dominant geometric variable (e.g., flute height). The plot reveals coupling between stiffness contrast and geometry.

Material contrast between liners and fluting layers, typically expressed via shear-modulus ratios, significantly affects the shear correction factor. The dependence of $k_{s}$ on modulus contrast is illustrated in Figure 6.

**Figure 7:**
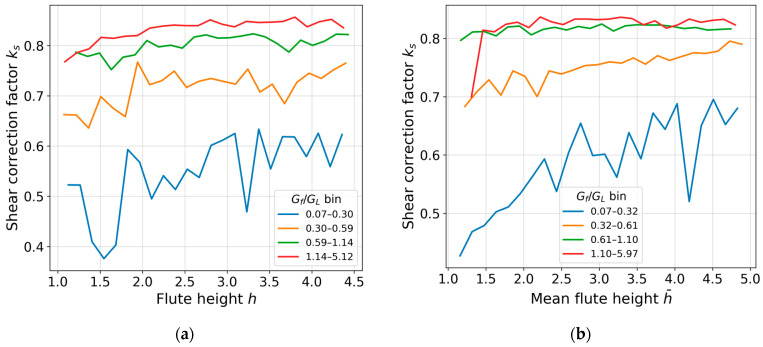
Interaction plot: $k_{s}$ versus flute height h for several bins of $G_{f}/G_{L}$ for (**a**) 3-ply and (**b**) analogous binned plots for 5-ply. The figure illustrates whether stiffness contrast amplifies or suppresses the geometric influence.

Interaction effects between material contrast and geometry are further examined using binned plots shown in Figure 7.

### 3.6. Comparison Between Three-ply and Five-ply Sensitivity Patterns

**Figure 8:**
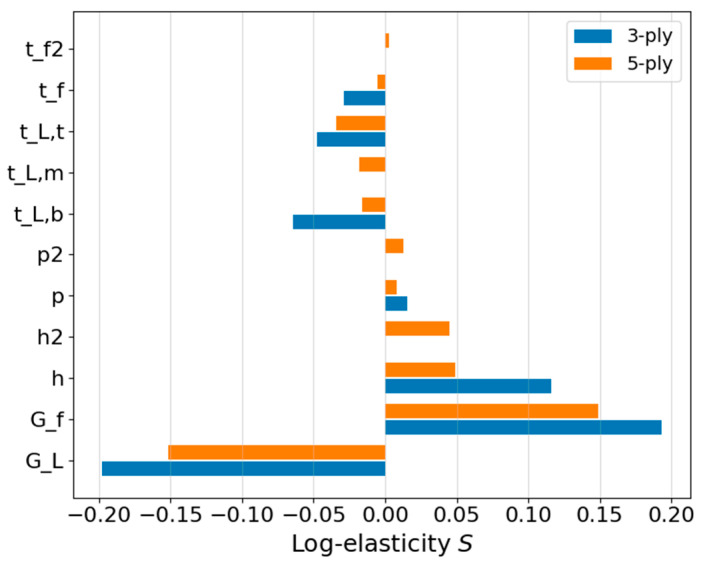
Side-by-side comparison of normalized sensitivities (log-elasticities) for 3-ply and 5-ply. Shared dominant drivers are highlighted, and configuration-specific drivers are identified.

A direct comparison of normalized sensitivities for the three-ply and five-ply configurations is presented in Figure 8, allowing universal and configuration-specific drivers of $k_{s}$ to be identified.

## 4. Discussion

The present study provides a comprehensive, global assessment of the shear correction factor $k_{s}$ for multiwall corrugated structures, combining large-scale parametric sampling with an energy-consistent, pixel-based formulation. By systematically analyzing both three-ply and five-ply configurations, the results allow for the clear identification of universal drivers, configuration-specific effects, and key geometric–material interactions governing transverse shear behavior.

### 4.1. Reliability of the Dataset and Numerical Robustness

A prerequisite for meaningful sensitivity analysis is numerical robustness. The adopted parameter ranges and sampling strategy, summarized in Table 1, ensure broad coverage of realistic corrugated board configurations while maintaining physical feasibility. The pixel-based geometries illustrated in Figure 1 confirm that the discretization captures essential geometric features for both single- and double-flute systems.

The resolution sensitivity study reported in Table 2 demonstrates that the computed shear correction factors are only weakly affected by pixel refinement. The small median and upper-percentile deviations indicate that the global trends identified in subsequent analyses are intrinsic to the structural parameters rather than artifacts of discretization. This robustness is particularly important for the five-ply case, where increased geometric complexity could otherwise amplify numerical sensitivity. Importantly, pixel refinement does not alter the ranking of dominant geometric and material drivers identified in the global sensitivity analysis, confirming that the reported sensitivity patterns are resolution-independent within the investigated discretization range.

### 4.2. Global Variability of the Shear Correction Factor

The distributions of $k_{s}$ obtained from Latin Hypercube Sampling, shown in Figure 2, reveal substantial variability across the investigated parameter space. The statistical descriptors provided in Table 3 confirm that this variability is significantly larger for five-ply boards than for three-ply boards. This finding reflects the additional deformation mechanisms introduced by the second fluting and the middle liner, which increase both geometric freedom and interaction effects.

The broader dispersion and heavier tails observed in Figure 2 indicate that extreme combinations of geometry and material contrast can lead to shear correction factors far from classical reference values. This result directly challenges the common practice of adopting a single, constant shear correction factor for corrugated structures, especially for multiwall configurations.

### 4.3. Dominant Parameters and Normalized Sensitivity Patterns

The normalized sensitivity analysis provides a quantitative ranking of parameter influence in a unit-free form. The results reported in Table 4 (three-ply) and Table 5 (five-ply) show that geometric parameters associated with fluting shape consistently dominate the response. This conclusion is reinforced by the tornado plots in Figure 3, where flute height and period emerge as the primary drivers of $k_{s}$ across the global parameter space.

While log-elasticities and PRCC largely agree in their ranking, discrepancies for selected parameters suggest the presence of nonlinear or non-monotonic effects. Such behavior is expected in corrugated structures, where local changes in geometry can alter load paths and energy distribution in a non-proportional manner. Importantly, the sensitivity patterns differ between three-ply and five-ply boards, indicating that conclusions drawn from single-wall systems cannot be directly transferred to multiwall configurations.

### 4.4. Physical Interpretation Via Response Surfaces

For the three-ply configuration, the response surface shown in Figure 4 reveals strong nonlinear coupling between flute period and height. Regions of high $k_{s}$ correspond to geometries that promote more uniform shear strain distribution across the cross-section, whereas low values are associated with slender or widely spaced flutes that concentrate shear deformation.

The five-ply response surfaces in Figure 5 further demonstrate that each fluting family contributes distinctly to the global shear response. Fixing one flute while varying the other reveals asymmetric influence patterns, highlighting the role of inter-layer interactions. These results confirm that the effective shear correction factor in multiwall boards cannot be interpreted as a simple superposition of individual flute contributions.

These response surfaces confirm the presence of non-additive coupling effects between geometric parameters and between geometry and material contrast, indicating that the shear correction factor cannot be expressed as a separable function of independent variables.

### 4.5. Role of Material Contrast and Interaction Effects

Beyond geometry, material contrast between liners and fluting layers plays a critical role in shaping transverse shear behavior. The scatter plots in Figure 6 show that $k_{s}$ varies systematically with shear-modulus ratios such as $G_{f}/G_{L}$, but the relationship is strongly modulated by geometry. This observation underscores that material contrast alone is insufficient to predict shear correction behavior without considering geometric context.

The interaction plots in Figure 7 provide further insight by demonstrating how stiffness contrast amplifies or suppresses geometric effects. For certain modulus ratios, changes in flute height lead to pronounced variations in $k_{s}$, while for others the response becomes comparatively insensitive. Such interactions explain the partial discrepancies observed between log-elasticities and PRCC in Table 4 and Table 5, and they emphasize the need for multidimensional analysis when assessing corrugated board shear behavior.

### 4.6. Comparison Between Three-ply and Five-ply Systems

A direct comparison of normalized sensitivities, presented in Figure 8, highlights both universal and configuration-specific drivers of the shear correction factor. Flute geometry emerges as a universal determinant of $k_{s}$ across both systems, confirming its fundamental role in transverse shear deformation.

However, the five-ply configuration exhibits additional influential parameters associated with the middle liner and the second fluting layer. These configuration-specific effects lead to higher variability and more complex interaction patterns, as already suggested by the broader distributions in Figure 2 and the response surfaces in Figure 5. Consequently, design rules or simplified models calibrated for three-ply boards may be inadequate or even misleading when applied to multiwall corrugated structures.

### 4.7. Implications for Modeling and Design

Taken together, the results presented in Figure 1, Figure 2, Figure 3, Figure 4, Figure 5, Figure 6, Figure 7 and Figure 8 and Table 1, Table 2, Table 3, Table 4 and Table 5 demonstrate that the shear correction factor for corrugated boards is not a fixed material constant but a highly configuration-dependent quantity. The strong sensitivity to geometry, material contrast, and their interactions implies that simplified assumptions, such as adopting a universal shear correction factor, can lead to significant modeling errors, particularly for multiwall systems.

The proposed energy-consistent, pixel-based framework provides a systematic means of quantifying these effects and can serve as a foundation for reduced-order models or design-oriented correlations. Importantly, the distinction between universal and configuration-specific drivers identified in Figure 8 offers guidance for developing simplified yet physically grounded design rules for corrugated structures of varying complexity. The present work is restricted to a computational sensitivity framework. Experimental validation of transverse shear stiffness in corrugated systems has been reported in previous studies [10,41,47] and is outside the scope of this global parametric investigation.

## 5. Conclusions

This study presented a global sensitivity analysis of the shear correction factor $k_{s}$ for three-ply and five-ply corrugated board configurations using an energy-consistent, pixel-based framework combined with Latin Hypercube Sampling.

The results demonstrate that the shear correction factor is not a fixed material constant, but a configuration-dependent quantity governed primarily by fluting geometry. In both single-wall and double-wall systems, flute height and period emerge as the dominant drivers of $k_{s}$, exceeding the influence of thickness and material parameters.

The five-ply configurations exhibit significantly broader variability and more complex interaction patterns than three-ply boards. The presence of two fluting layers and an intermediate liner introduces non-additive coupling effects, meaning that the effective shear behavior cannot be interpreted as a simple superposition of independent layer contributions.

Material contrast between fluting and liners systematically affects $k_{s}$, but its influence is strongly modulated by geometry. In certain geometric regimes, stiffness contrast amplifies shear sensitivity, while in others it has a comparatively limited effect. This confirms that geometry–material interactions must be considered jointly in reduced-order modeling.

The findings highlight that adopting a single, constant shear correction factor for corrugated structures—particularly for multiwall boards—may lead to significant inaccuracies. The proposed framework enables configuration-dependent identification of $k_{s}$ and provides a systematic basis for robust reduced-order modeling of periodic-core structures.

## Figures and Tables

**Table 1:** Parameter ranges used in the LHS sensitivity study for 3-ply and 5-ply corrugated board. All moduli are sampled log-uniformly; geometric parameters are sampled uniformly. The minimum thickness constraint $t_{\min}\geq 2$ pix is enforced.

Parameter	3-ply		5-ply	
	Min	Max	Min	Max
p [mm]	4.000258	8.499252	4.002924	9.494775
h [mm]	1.001079	4.497692	1.001209	4.998968
$t_{f}$ [mm]	0.100412	0.349547	0.100012	0.349896
$p_{2}$ [mm]	-	-	4.001369	9.498377
$h_{2}$ [mm]	-	-	1.000964	4.997345
$t_{f2}$ [mm]	-	-	0.100056	0.349870
$t_{L,t}$ [mm]	0.100053	0.399727	0.100234	0.399820
$t_{L,m}$ [mm]	-	-	0.100245	0.399957
$t_{L,b}$ [mm]	0.100311	0.399791	0.100196	0.399823
$G_{f}$ [MPa]	0.150515	1.493291	0.150255	1.496948
$G_{L}$ [MPa]	0.250567	2.498199	0.250230	2.499512

**Table 2:** Resolution robustness check: summary statistics of $\Delta k_{s}/k_{s}$ (in %) when refining pixel size for a subset of randomly selected samples. Reported are median, 90th percentile, and maximum relative changes.

Dataset	Median [%]	90th percentile [%]	Max [%]
**3-ply**	0.165672	1.853076	7.497941
**5-ply**	0.023637	1.082396	7.299894

**Table 3:** Summary statistics of $k_{s}$: mean, standard deviation, median, interquartile range (IQR), and selected quantiles (5%, 50%, 95%) for 3-ply and 5-ply datasets.

Dataset	Mean	std	Median	IQR	q5	q50	q95
3-ply	0.718413	0.128947	0.760203	0.176524	0.466396	0.760203	0.849355
5-ply	0.741803	0.112452	0.787480	0.123227	0.482342	0.787480	0.838569

**Table 4:** Normalized global sensitivity indices for 3-ply: log-elasticities and PRCC for each input parameter. Positive values indicate an increase of $k_{s}$ with increasing parameter, negative values indicate a decrease.

Parameter	Log-Elasticity	PRCC
p	0.015330	0.074295
h	0.115772	0.530662
$t_{f}$	−0.029048	−0.171102
$t_{L,t}$	−0.047826	−0.142491
$t_{L,b}$	−0.064391	−0.195491
$G_{f}$	0.023640	0.093608
$G_{L}$	−0.010947	−0.056239

**Table 5:** Normalized global sensitivity indices for 5-ply: log-elasticities and PRCC for each input parameter.

Parameter	Log-Elasticity	PRCC
$p_{1}$	0.008090	0.019066
$h_{1}$	0.049138	0.157244
$t_{f1}$	−0.005123	0.063589
$p_{2}$	0.012620	0.003505
$h_{2}$	0.044675	0.144208
$t_{f2}$	−0.009513	0.061353
$t_{L,t}$	−0.018829	−0.080112
$t_{L,m}$	−0.031460	−0.125244
$t_{L,b}$	−0.021187	−0.091543
$G_{f}$	0.019820	0.072902
$G_{L}$	−0.006341	−0.031775

## Data Availability

The original contributions presented in this study are included in the article. Further inquiries can be directed to the corresponding author.
